# A Review of Vaccine Approaches for West Nile Virus

**DOI:** 10.3390/ijerph10094200

**Published:** 2013-09-10

**Authors:** Arun V. Iyer, Konstantin G. Kousoulas

**Affiliations:** Division of Biotechnology and Molecular Medicine, Louisiana State University School of Veterinary Medicine, Skip Bertman Drive, Baton Rouge, LA 70803, USA

**Keywords:** West Nile Virus, vaccine, flaviviruses, envelope glycoprotein, WNV lineage, veterinary vaccine

## Abstract

The West Nile virus (WNC) first appeared in North America in 1999. The North American lineages of WNV were characterized by the presence of neuroinvasive and neurovirulent strains causing disease and death in humans, birds and horses. The 2012 WNV season in the United States saw a massive spike in the number of neuroinvasive cases and deaths similar to what was seen in the 2002–2003 season, according to the West Nile virus disease cases and deaths reported to the CDC by year and clinical presentation, 1999–2012, by ArboNET (Arboviral Diseases Branch, Centers for Disease Control and Prevention). In addition, the establishment and recent spread of lineage II WNV virus strains into Western Europe and the presence of neurovirulent and neuroinvasive strains among them is a cause of major concern. This review discusses the advances in the development of vaccines and biologicals to combat human and veterinary West Nile disease.

## 1. Introduction

West Nile virus (WNV) belongs to the flavivirus genus in the family Flaviviridae and was first isolated in 1937 from a febrile patient in the West Nile province of Uganda [1]. WNV has an approximately 11 kb single-stranded positive (+) sense RNA genome. The virions are icosahederal particles about 500 Å in diameter and have 180 molecules of pre-membrane/membrane (preM/M) and 180 molecules of envelope (E) glycoprotein on its surface [2]. The genome is translated as a single polyprotein, which is subsequently cleaved by host and viral proteases producing three structural and seven nonstructural proteins [3,4]. The structural proteins include a capsid (C) protein, a premembrane (prM) protein, and an envelope (E) glycoprotein that mediates attachment, virus-induced membrane fusion and virion assembly [5,6]. PreM (26 kD) is one of the two glycoproteins expressed on the viral surface and plays a critical role in E glycoprotein folding and chaperoning [7,8]. The PreM also functions in protecting the E glycoprotein from the intracellular acidic environment, which can cause structural rearrangement to a fusogenic form, as the heterodimeric preM/E complex passes through the secretory pathway. The WNV E glycoprotein is an approximately 53 kD type I membrane glycoprotein and has 12 conserved cysteine residues [9]. The E glycoprotein has three domains (DI-DIII). DI is the central structural domain while DII is the dimerization domain. The DII domain is made up of a 12 amino acid (aa) long fusion loop, which is necessary for virus-cell membrane fusion and for receptor binding [10]. The DIII domain spans amino acids 296–415 and functions in receptor recognition and binding [11,12,13,14,15]. 

More importantly, from a vaccinologists’ perspective, a majority of the neutralizing epitopes has been mapped to DIII [11,16,17,18,19,20]. The residues most consistently identified as neutralization epitopes on DIII are amino acids 302–309, 330–333, 333–365 and 389–391 [18]. Co-crystallization studies of DIII with monoclonal antibody mAbE16 revealed that these regions mapped to adjacent Ig-like loops forming a discontinuous epitope [17]. Potent monoclonal antibodies can block WNV infection even at low occupancies by binding to as few as 30 of the 180 envelope proteins on the virion [21].

Phylogenetic data of WNV reveals at least two distinct lineages [22,23,24,25]. Strains from North America, Europe, Middle East, Australia and India belong to lineage I. The Indian, Australian (Kunjin), Czech (Rabensburg) and LEIV-Krnd88-190 virus (Russia) isolates form separate sub-lineages within lineage I [22,25,26,27,28]. The Rabensburg, Russian and Indian isolates have been classified as separate lineages *viz*. III, IV and V by some researchers [24,25,29,30], while the divergent Kunjin virus from Sarawak and the African Koutango virus have been classified as lineages VI and VIII [30], respectively. Lineage II WNV was mainly restricted to sub-Saharan Africa and the island of Madagascar until it established itself in Hungary in 2004 [29,31]. 

Following its initial isolation in 1937 in Uganda [1], WNV outbreaks were reported in Egypt and Israel (1951 and 1957), France (1962–1965), South Africa (1974), India (1980-1981) and the Ukraine (1985). Also, WNV outbreaks have occurred in the recent past in Algeria (1994), Romania (1996), Morocco (1996 and 2003), Tunisia (1997 and 2003), Italy (1998), Czech Republic (1997), Israel (1997, 2000 and 2003), Russia (1999–2001), and France (2000, 2003–2004) [32,33,34,35,36,37,38,39,40,41,42,43,44,45,46]. 

WNV was not considered a major risk for humans until the 1996 outbreak in Romania. However, WNV subsequently became a major veterinary and public health concern in Europe and United States. The first cases of West Nile (WN) infections in the United States were reported in Flushing , NY in 1999 [47]. The more recent outbreaks include the 2008–2009 season in Italy [48] resulting in 17 cases of WN neuroinvasive disease, the 2010 outbreak in Greece [49] with a whopping 17% mortality rate and the 2011 Kunjin virus outbreak among horses in Australia [50].

Since its emergence in Hungary in 2004 [29,31], the lineage II viruses have recently appeared in other non-traditional geographical zones. Retrospective studies have shown that that the lineage 2 WNV circulated among birds in Austria in 2008/2009 [51]. The Lineage II WNV has also been responsible for major outbreaks in Russia [52], Italy [53] and Greece [54]. Complicating matters further, highly neuroinvasive lineage II viruses have been isolated and have been shown to co-circulate along with lineage I viruses [55]. A fatal lineage II WNV case of an elderly Belgian national returning from Greece was recently described highlighting travel-associated transmission [56]. Weather patterns, virus adaptation to local vectors along with natural bird-migration related transmission of the virus could aid the successful establishment of the virus in Europe. It is now evident that neuroinvasive and neuropathogenic strains of WNV exist among both the lineage I and II WNV and their emergence, transposition and adaptation to new locales pose significant threats to humans, horses and birds. 

## 2. WNV in Mosquitoes, Birds and Horses

WNV is primarily transmitted in nature by *Culex* mosquitoes. However, the virus has been isolated from more than 60 species of mosquitoes, [57,58] as well as from ticks [59,60]. Mosquito saliva is known to greatly enhance the success of virus infection by temporarily suppressing the host immune system in multiple ways [61]. A study comparing four species of mosquitoes showed that each mosquito was able to inject a mean dose of 10^3.6–5.9^ Plaque Forming Units (PFU) of WNV per bite [62]. Additionally, approximately 10^2^ PFU of WNV was shown to be injected extravascularly [62]. Mosquitoes become infected with WNV as they imbibe a blood meal from a viremic host. They can also infect each other as they simultaneously feed on the same host [63]. The virus infects the gut epithelium cells of the mosquitoes and as viral titers increase, they enter the hemocoel, spread to surrounding tissues, and finally invade the salivary glands and brain of the mosquito vector [64,65]. When the infected mosquito bites a vertebrate, WNV is transmitted intradermally. The virus infects and replicates in the Langerhans cells of the host. As the Langerhans cells migrate to the lymph nodes, a second round of replication takes place in the lymph nodes before the onset of viremia [66]. 

Birds are amplifiers of the disease. The North American strain of WNV is characterized by its ability to cause fatal neurological disease among many different species of birds. WNV has been isolated from at least 300 species of birds and has been shown to cause specific pathological changes in many tissues of at least 14 different species [45,64,67]. Thus, birds are the most important link in WNV maintenance and transmission. American alligators (*Alligator mississippiensis*) can also become infected with WNV and transmit WNV to mosquitoes [68,69]. Additionally, WNV has been isolated from a number of non-avian vertebrate hosts including, but not limited to equine, feline and canine hosts in addition to bats [64,70]. Like birds, horses play an import indicator role in WNV outbreaks but are dead-end hosts. They become viremic but the viremia is not high enough for horses to serve as an amplifying host [71]. In the 1999 outbreak, 36% of the infected horses either died of WN or were euthanized [72]. Other studies reported a 22–28% mortality rate in horses [73,74]. Lineage II WN viruses were associated with a 70% (five out of seven animals) mortality rate in horses in South Africa [75]. The common clinical symptoms in horses include weakness, incoordination and ataxia [76].

## 3. Human West Nile Disease

WNV is a Category B National Institute of Allergy and Infectious Diseases (NIAID) Priority Pathogen [77]. It has also been a nationally notifiable disease since 2005 [78]. 

Humans are dead-end hosts for WNV as the virus does not achieve sufficiently high enough titers to be transmitted to mosquitoes. WNV has also been reported to be transmitted to humans through non-vector mediated routes. WNV can be transmitted from mother to child by the intrauterine route [79], through breast milk [80,81] and blood transfusion [82,83,84]. Additionally, the virus can be transmitted via bone marrow transplant [85], organ transplantation [86,87] and through dialysis [88,89]. Laboratory acquired infections have also been reported [90,91].

The incubation period for human WNV disease is between 2–14 days [92]. Approximately 80% of patients infected with WNV are asymptomatic and 20% of the patients suffer from West Nile Fever (WNF); a condition that is characterized by fever and headache [92,93,94]. Additionally, some patients may also exhibit a variety of signs and symptoms including muscle weakness, fatigue, nausea, vomiting, gastrointestinal problems, lymphadenopathy and non-pruritic maculopapular skin rash [65,95,96]. Non-neurological clinical manifestations of WNF include rhabdomyolysis [97,98], pancreatitis [99], hepatitis [100], myosistis, orchitis [101], chorioretinitis [102] and cardiac dysrythmias [103]. 

Less than 1% of the WNV patients suffer from a West Nile Neuroinvasive Disease (WND) including West Nile meningitis (WNM), encephalitis (WNE) and acute flaccid paralysis (a poliomyelitis-like syndrome) (WNP) [93,103,104]. The differences in symptoms of WNF and WND are often difficult to distinguish. WNM is believed to occur in approximately 40% of the cases of WND. Symptoms of meningeal irritation including fever, headache, nuchal rigidity, photophobia, and phonophobia have been observed in these patients. Some of the patients also exhibit Kernig’s and Brudzinski’s signs [105]. Pleocytosis of <500/mm^3^ is often observed [106]. The prognosis for recovering from WNF and WNM is generally favorable with occasional complaints of weakness and issues relating to memory and concentration on follow-up examination [107]. 

A second aspect of WND is WNE. The severity of WNE can range from mild encephalitic disease to a more severe from characterized by coma and death and the risk of developing WNE increases with age [108]. Patients with WNE exhibit depression, altered level of consciousness or confusion and personality changes [104,109]. Additional symptoms include ataxia, lethargy, movement disorders, Parkinsonism, conjunctivitis, confusion, photophobia, slurred speech, seizures, tremors and involuntary body movements [65,66,103,104,110,111]. Prognosis for WNE patients is not as good as those for WNM with some patients suffering from functional and cognitive problems [107]. Interestingly, WNE was first reported in the United States in 1952 in New York when the Egyptian strain of WNV was used in treatment of cancer. Encephalitis was observed in 9.47% (nine out of 95) of these patients [112]. 

West Nile poliomyelitis (WNP) is the third aspect of WND and is the result of WNV infecting the motor neurons. This results in asymmetric acute flaccid paralysis of one or more limbs [97,106,113,114]. Some patients may have to be put on mechanical ventilation and intubation as the diaphragmatic and intercostal muscle may be paralyzed causing respiratory failure [103,114,115]. Areflexia or hyporeflexia, loss of bowel and bladder control are also common [103]. A Guillain-Barré-like syndrome affecting peripheral nerves, radiculopathy and demylenating peripheral neuropthay have also been reported although true Guillain-Barré syndrome is rare [106,115,116,117]. Recovery was found to be variable and in general, the initial severity of the disease did not forebode a poor prognosis [106,118].

Among WND human cases, an estimated 55–60% patients suffer from WNE with an estimated 20% case fatality. Additionally, WNP may contribute to 10–50% case mortality in humans [104]. The most common victims of WNV are the very young, the elderly and those with suppressed or compromised immune systems. 

## 4. Advances in Vaccine Approaches against West Nile Virus

WNV was first isolated more than seventy years ago [1]. It was not considered to be a worrisome flavivirus until it invaded North America causing disease with neurological implications. Currently, there are no commercially available vaccines for human use. Several vaccines and biologicals for human use have been evaluated in Phase I and Phase II clinical trials in the US [119]. A major reason for not developing a human WNV vaccine is probably the lack of substantial commercial interest. There is a very small and practically economically insignificant market for a human vaccine. The prohibitively high cost-to-benefit ratio for development and marketing is the primary cause for preventing its development and commercialization [120]. In this context, Crucell Inc. launched an initiative to develop a WNV vaccine for human use, but later discontinued its efforts [121]. 

In contrast to the lack of WNV vaccines for humans, a number of experimental vaccines have been successfully developed and tested, and several vaccines have been licensed for veterinary use. A wide number of vaccine approaches have been tested in mice, hamsters, birds, horses and non-human primates. Some human vaccine candidates have been evaluated in phase I and II clinical trials.

### 4.1. Inactivated Vaccines

A formalin-inactivated whole-virus veterinary vaccine originally developed by Fort Dodge Animal Health, Fort Dodge, IA, USA was licensed in 2003 (marketed by Pfizer under the brand name West Nile Innovator^®^). This vaccine was shown to be safe and efficacious in horses and was granted full license by the USDA [122]. In small animal experiments, golden hamsters vaccinated with the Fort Dodge vaccine were completely protected against challenge. Almost 89% of the vaccinated animals showed hemagglutination inhibition (HI) and complement fixing (CF) antibodies while 55.5% animals showed low levels of WN neutralizing antibodies [123]. The vaccine, however, could not efficiently elicit neutralizing antibodies in flamingos and red-tailed hawks [124]. The Innovator^®^ vaccine was also tested in the baboon model for WN. Vaccinated baboons showed increased IgG and IgM response and high PRNT titers [125]. The animals exhibited very low viremia on challenge with WNV-OK2 strain.

An inactivated equine WNV vaccine was tested on horses in 2003 [122]. On challenge, 81.8% of the control horses had viremia as compared to 19% of the vaccinated horses. An experimental vaccine using formalin inactivated WNV passaged in sucking mouse brains was evaluated in young geese. Eighty-five percent of the birds were protected upon intra-cranial challenge with WNV. Extensive field studies showed the vaccine was safe and efficacious [126]. The same research group also developed an inactivated vaccine using the PER.C6 cell platform. They showed that 91.4% of the vaccinated geese were protected following severe intracranial challenge [127]. Boehringer Ingelheim Vetmedica markets a USDA licensed killed virus vaccine under the trade name Vetera^TM^ WNV. Recently, Diamond et al. showed that young and aged mice vaccinated and boosted with 3% hydrogen peroxide inactivated Kunjin WNV elicited a 90–100% protective immune response following lethal intracranial challenge [128]. 

### 4.2. Recombinant/Subunit Vaccines

The WNV envelope glycoprotein gene derived from a mosquito isolate was used to express the E glycoprotein. C3H/HeN mice vaccinated with the purified protein were shown to be protected on challenge [129]. In another experiment, soluble WNV E protein expressed in S2 cells was used to vaccinate mice and horses. All vaccinated mice survived challenge and both the mice and the horses developed high titer WN antibodies [130]. Watts *et al.* [131] immunized hamsters with a carboxy terminus-truncated WNV E protein in combination with or in absence of the WNV non-structural protein 1 (NS1). Animals in the NS1-only group showed an 87% survival rate, whereas animals vaccinated with NS1 and E or just E alone showed a 100% survival rate on challenge. In a follow-up study by the same research group, the authors reported robust cellular immune responses in vaccinated hamsters [132]. 

Domain III (D III) of the flavivirus envelope gene is highly immunogenic. Researchers have successfully made subunit vaccines for Dengue [133] and Japanese encephalitis [134] using the D III region. In 2007 Chu *et al*. [20] evaluated a WNV DIII based vaccine for WNV and showed that it elicited a strong Th1 response with production of IL-2 and IFN-γ. McDonald *et al.* generated a WNV EIII domain-bacterial flagellin (STF2∆) fusion protein [135]. This vaccine was able to stimulate both innate and adaptive immune responses and protected mice against challenge. A subunit vaccine expressing the D III of WNV E gene was evaluated in mice [136]. All vaccinated mice survived challenge as compared to an 80% survival rate observed in a β-propiolactone inactivated whole virus vaccine group. 

Recently, our group at Louisiana State University constructed a subunit vaccine by fusing the DIII domain of the highly neuroinvasive WNV LSU-AR01 [137] to equine CD40L. Our data showed that the vaccine induced strong neutralizing antibody responses in horses as measured by plaque reduction neutralization test (PRNT) after a single vaccination. A booster shot enhanced the antibody response. The vaccine was safe with no injection site reactions [138]. 

### 4.3. Nucleic Acid/DNA Vaccines

A DNA vaccine expressing the WNV NY99 capsid gene was constructed and tested in mice [139]. This vaccine was shown to elicit a strong Th1 immune response with a robust peak in IL-2 and IFN-γ levels. Davis *et al.* [140] engineered a DNA vaccine (pCBWN) expressing WNV preM and E proteins. The vaccine protected 100% of the mice and generated robust neutralizing antibody response in horses on challenge. The pCBWN vaccine was also shown to protect American crows (*Corvus brachyrhynchos*) and fish crows (*Corvus ossifragus*) in a route dependent manner [141,142]. A plasmid DNA encoding the infectious full-length RNA genome of Kunjin virus was used to vaccinate mice. A single mutation in the NS1 gene of the Kunjin virus attenuated it in sucking mice. The vaccine was shown to protect against intracerebral and intraperitoneal challenge with both WNV NY99 and the Kunjin virus [143]. Phase I clinical trials for a DNA vaccine expressing WNV NY99 preM and E genes were conducted in 2007 [144]. This vaccine was found to be safe and well-tolerated and elicited strong humoral and T cell response. A very similar vaccine with a modified CMV/R promoter was recently evaluated in a Phase I clinical trial. This vaccine was shown to be safe and elicited neutralizing antibody response even in older individuals [145]. 

More recently, a capsid deleted Kunjin virus DNA vaccine was developed with the capsid being provided in *trans*. These single-cycle viruses replicate once to generate VLPs, which were highly immunogenic in mice and horses [146]. The WNV innovator-DNA developed by Fort Dodge Animal Health became the first DNA vaccine licensed by the USDA for use in horses in 2005, but was later removed from the market [57]. This vaccine was also shown to be one of the two best vaccine options in protecting scrub jays (*Aphelocoma sp.*) [147].

### 4.4. Recombinant Virus Vaccines

The recombinant live canarypox vectored vaccine by Merial (Sanofi) was licensed in 2004 for veterinary use. This vaccine marketed under the brand name RecombiTEK has a canapox/ALVAC viral vector backbone expressing WNV preM and E proteins. The RecombiTEK vaccine was shown to elicit a strong anamnestic immune response in horses that were previously vaccinated with the Fort Dodge Innovator^®^ vaccine [148]. A single dose of the vaccine afforded early protection against viremia in horses challenged with WNV infected mosquitoes [149]. When vaccinated with two doses and subjected to a mosquito challenge, all vaccinated horses developed high titer neutralizing antibody response and did not show any clinical signs of illness [150]. The RecombiTEK vaccine has also been proven to be effective in cats [151]. In a separate set of experiments, ten control and ten RecombiTEK vaccinated horses were challenged by the intrathecal route and was shown to be protective considering the challenge route [152]. More significantly, this vaccine was recently shown to protect horses from neurovirulent lineage 2 WNV [31].

A lentivirus vector based vaccine (TRIP/sE_WNV_) was tested in mice. A single dose of this vaccine protected against lethal challenge with WNV IS-98-ST1 strain. This protection was seen as early as seven days post vaccination and also provided long lasting immunity [153]. 

A live measles virus vaccine expressing secreted envelope glycoprotein of the IS-98-ST1 strain of WNV was constructed using the attenuated Schwarz strain of measles virus. This vaccine (MVSchw-sE_WNV_) protected mice against lethal challenge with WNV [154]. Recombinant equine herpes virus-1 (EHV-1) vectored vaccines expressing WNV PrM and E proteins was shown to elicit neutralizing antibody response in horses as measured plaque reduction neutralization test [155].

We constructed and tested in mice recombinant vesicular stomatitis virus (VSV) vectored vaccines against WNV using a prime-boost approach. The envelope glycoprotein from the highly neurovirulent LSU-AR01 [137] strain was cloned into recombinant VSV that expressed either VSV Indiana G glycoprotein (priming vaccine) or Chandipura virus G glycoprotein (boosting vaccine). The vaccine protected 90% of the vaccinated mice against severe lethal challenge with LSU-AR01 virus. In addition, the vaccine elicited robust WNV E glycoprotein specific cellular immune response including upregulation of antigen specific CD4^+^CD154^+^IFNγ^+^ T cells and CD8^+^CD62L^low^ IFNγ^+^ T cells [156].

### 4.5. ChimeriVax Technology Based Vaccines

To obtain license for a commercial West Nile vaccine for human use, the vaccine must demonstrate safety and efficacy in clinical trials. Importantly, the vaccine must be able to elicit potent protective immune responses. The ChimeriVax vaccine in many ways exploits the clinical data that exists for its parent vaccine the Yellow fever 17D vaccine. The vaccine virus, known as the Asibi strain, was isolated from a patient named Asibi in Ghana in 1927 [157,158]. In 1930, Max Theiler developed the first attenuated strain of this virus which he called the 17D virus [157]. The vaccine has demonstrated a very good safety record of millions of doses over the years [120,159]. The ChimeriVax vaccines largely rely on their comparative safety against this vaccine. The ChimeriVax vaccines have a vector backbone consisting of the 17D non-structural genes. The preM and E genes of the candidate flavivirus is incorporated into this backbone generating a recombinant virus expressing the antigens of interest in a 17D background.

The first chimeras contained the Japanese encephalitis (JE) virus preM and E [160], were shown to be genetically stable and conferred strong protection against virulent JE virus challenge [161]. The ChimeriVax^TM^-JE virus did not infect *Aedes* or *Culex* mosquitoes [162]. This vaccine has been extensively tested and characterized in mice [161] and Rhesus macaques [163,164]. ChimeriVax^TM^-JE has been studied in humans and a Phase II clinical trial its potential effectiveness as a human vaccine [165,166]. Pugachev *et al.* [167] have published a detailed review on these vaccines. Similar vaccines have also been generated and tested for all four Dengue virus serotypes [168,169,170,171,172,173]. The ChimeriVax^TM^-Dengue vaccine was evaluated in Phase II clinical trials [174].

Studies in the hamster model showed that the ChimeriVax^TM^-WNV protected hamsters and induced a strong immune response as measured by HI, CF and the plaque reduction neutralization test (PRNT) [123]. Preclinical studies were also carried out in mice and Rhesus macaques. ICR mice that were vaccinated and challenged intraperitoneally were protected in a vaccine dose-dependent manner. Similarly, ChimeriVax^TM^-WNV vaccinated macaques were uniformly protected against intracerebral challenge [175]. About 50% of these animals suffered from subclinical disease post challenge and this is attributed to the aggressive route of challenge. In a second set of pre-clinical studies, the ChimeriVax-WNV_02_ vaccine, which has multiple point mutations, was tested in rhesus macaques. The skin and lymphoid tissues were prominent sites for viral replication. Additionally, studies on human subjects revealed that the vaccine produced high titer neutralizing antibody response and antigen specific CD8^+^ T cells producing IFN-γ [176]. WNV specific CD4^+^ T cells were detected in >80% of the subjects. In Phase II clinical trials, the ChimeriVax-WNV vaccine was evaluated in studies based on age and dose. The vaccine was well tolerated and immunogenic in younger adults (18–40 years), patients 50 years of age or older and in patients over 65 years of age [177,178].

An equine WNV vaccine based on the ChimeriVax technology was licensed by the USDA in 2006 and marketed by Intervet under the brand name Prevenile. This vaccine encoded the WNV NY 99 pre-membrane and envelope genes on a YFV backbone, but was recalled in 2010 due to acute side effects including fatality among vaccinated horses [179].

### 4.6. Virus-Like Particles (VLP) and Heterologous Vaccines

A number of other approaches have been used to produce effective WN vaccines. Qiao *et al.* [180] generated WN virus-like particles (WNVLPs) using recombinant baculovirus expressing a combination of preM and E genes or capsid, preM and E genes. WNVLPs expressing preM and E generated a strong, protective and sterilizing immunity in mice on challenge. However, when the capsid protein was included to generate WNVLPs, a much weaker immune response was observed.

Heterologous vaccine approaches have been used to develop WNV vaccines based on cross protection afforded by closely related flaviviruses. An example of this approach is the use of the Israel turkey meningoencephalitis virus (ITMV). ITMV is an arbovirus belonging to Ntaya serogroup of flaviviruses and was first described in 1960 [181]. It was found that turkeys less than ten weeks seldom showed any incidence of WNV probably due to presence of cross reacting antibodies. Geese vaccinated with formalin inactivated ITMV vaccine exhibited 39–72% survival on intracranial challenge with WNV [182]. In 1971, Price and Thind [183] demonstrated that hamsters vaccinated with any of four Dengue virus serotypes were protected against a WNV challenge. Hamsters vaccinated with Japanese encephalitis virus vaccine (JEV SA14-2-8), wild-type St. Louis encephalitis virus (SLEV), or Yellow fever virus vaccine (YFV 17D), showed some level of protection against WNV. Animals vaccinated with the JEV SA14-2-8 and the SLEV vaccine were protected against WNV encephalitis and death [184]. American crows (*Corvus brachyrhynchus*) vaccinated with wild-type Kunijin virus were completely protected against WNV challenge [185] (the Kunjin viruses are now classified as lineage(s) of WNV).

Despite the encouraging results with cross-protection through the use of heterologous flavivirus vaccines, it is known that humans vaccinated with the JE vaccine (JE-VAX, BIKEN, Japan) or with Dengue vaccine (Aventis Pasteur, Lyon, France) do not show protective neutralizing antibodies against WNV [186]. Similar observations were made by Takasaki *et al.* [187] revealing that mice vaccinated with mouse brain-derived JE vaccine were not protected against intracranial challenge with WNV. However, at higher vaccine doses, animals were partially protected when challenged through intraperitoneal route. Interestingly, mice vaccinated against WNV elicited partial protective response against JEV [136]. 

### 4.7. Passive Antibody Prophylaxis

Hyperimmune sera have been used for passive antibody prophylaxis for many diseases including West Nile. Pooled sera from mice that were actively immunized with WNV E protein was diluted 1:5 and administered to naïve mice. These mice were challenged with 10^1^–10^6^ PFU WNV after 24 h of passive immunization. Four out of five control mice and one out of five vaccinated mice died over a 15 day observation period [129]. In one study immunocompetent and immunocompromised mice were administered polyclonal WN antibodies prior to infection with the virus. The antibody prevented morbidity in wild-type mice but the immunocompromised mice eventually succumbed at later time points [188]. Passively administered sera from immunized horses has been shown to protect naïve mice on challenge with WNV [130]. Similarly, affinity purified horse antibodies against three WNV envelope peptides protected 48–59% mice when challenged with WNV [189].

Passive antibody prophylaxis has been used with a fair amount of success in humans. Shimoni *et al.* [190] described a case of a 70 year old Israeli woman who went into deep coma in 72 h after admission. She was intravenously administered Omr-IgG-am antibodies (Omrix Biopharmaceutical Ltd, Kiryat-Ono, Israel) at 0.4g/Kg. The patients’ level of consciousness dramatically improved over the next week. In 2002, a 42 year old Israeli male lung transplant recipient with confirmed WNE exhibited deteriorating level of consciousness. Within 48 h of being intravenously administered Omr-IgG-am antibodies [191], the patient showed dramatic improvement. Passive antibody therapy, however, failed to protect a 55 year old man suffering from chronic lymphocytic leukemia, hypogammaglobulinemia and WNV infection. The patient was administered the Omr-IgG-am antibodies at 0.5 g/kg. Unfortunately, possibly because of the timing of administration and/or the underlying conditions, the patient succumbed thirty-two days into his illness [192].

### 4.8. Live-Attenuated Virus Vaccines

A live attenuated vaccine was generated by serially passaging the Israeli strain of WNV in a mosquito cell line and selecting an escape mutant using monoclonal antibodies [193]. The resulting virus, WN-25A lost all neuroinvasivenes, while it protected mice and geese upon lethal challenge. A couple of WNV and Dengue virus type 4 chimeras were constructed and evaluated as vaccines for WNV. In one chimera, the WNV membrane precursor and envelope were cloned on a Dengue 4 (WN/DEN4) background and the other had a 30 nucleotide deletion in the 3′ non-coding region of DEN4 (WN/DEN4-3′∆30). Both these vaccines were attenuated in Rhesus macaques and prevented viremia in the macaques upon challenge [194]. A follow-up study with the WN/DEN4-3′∆30 virus showed that it was unable to infect geese, and that it was safe in immunocompomised mice and attenuated in monkeys [195]. A similar study using a chimeric Dengue 2 virus construct expressing the WNV NY99 preM and E glycoprotein protected mice on challenge with the NY99 strain of WNV [196]. In another study, a molecularly cloned descendant of the lineage II prototype B956 was generated. This virus (WN1415), was shown to elicit a potent immune response and protect 100% of the mice on challenge, while at a lower vaccine dose (55 PFU), 67% of the mice were protected [197].

## 5. Conclusions

In conclusion, a number of vaccine strategies have been explored and showed promise to combat WNV. It is also evident that vaccines against lineage I WNV are able to protect against lineage II viruses and the converse is also true. In this regard, Kunjin virus-based vaccines are able to protect against lineage I viruses. A number of successful, licensed veterinary vaccines against WNV are readily available and several promising human WNV vaccine candidates have undergone successful early phase clinical trials. The spread of neurovirulent and neuroinvasive lineage II WNV through Europe is reminiscent of the invasion and spread of lineage I WNV through the North American continent. Licensed vaccines for human use are needed to combat WNV lineage I and potential lineage II world-wide infections.
